# Crash severity analysis: A data-enhanced double layer stacking model using semantic understanding

**DOI:** 10.1016/j.heliyon.2024.e30117

**Published:** 2024-04-29

**Authors:** Di Yang, Tao Dong, Peng Wang

**Affiliations:** a School of Computer Science and Technology, Changchun University of Science and Technology, Changchun, 130022, China; b Jilin Provincial Joint Key Laboratory of Big Data Science and Engineering, Changchun, 130022, China; c Chongqing Institute of Changchun University of Science and Technology, Chongqing, 401120, China

**Keywords:** Urban traffic crashes, Crash severity analysis, Semantic understanding, Data enhancement, Stacking model

## Abstract

The crash severity analysis is of significant importance in traffic crash prevention and emergency resource allocation. A range of innovations offers potential traffic crash severity prediction models to improve road safety. However, the semantic information inherent in traffic crash data, which is crucial in enabling a deeper understanding of its underlying factors and impacts, has yet to be fully utilized. Moreover, traffic crash data are commonly characterized by a small sample size, which leads to sample imbalance problem resulting in prediction performance decline. To tackle these problems, we propose a semantic understanding-based data-enhanced double-layer stacking model, named EnLKtreeGBDT, for crash severity prediction. Specifically, to fully leverage the inherent semantic information within traffic crash data and analyze the factors influencing crashes, we design a semantic enhancement module for multi-dimensional feature extraction. This module aims to enhance the understanding of crash semantics and improve prediction accuracy. Then we introduce a data enhancement module that utilizes data denoising and migration techniques to address the challenge of data imbalance, reducing the prediction model's dependence on large sample crash data. Furthermore, we construct a two-layer stacking model that combines multiple linear and nonlinear classifiers. This model is designed to augment the capability of learning linear and nonlinear mixed relationships, thereby improving the accuracy of predicting the severity of crashes on complex urban roads. Experiments on historical datasets of UK road safety crashes validate the effectiveness of the proposed model, and superior performance of prediction precision is achieved compared with the state-of-the-arts. The ablation experiments on both semantic and data enhancement modules further confirm the indispensability of each module in the proposed model.

## Highlights

• A double-layer Stacking model for crash severity prediction is proposed.
• A semantic enhancement module is designed to improve feature understanding.
• A data enhancement module is constructed to address data imbalance.
• The prediction precision increases 4.34% compared with the-state-of-arts.

## 1 Introduction

With the rapid urban growth, the proliferation of automobiles has led to an alarming rise in traffic crashes that have caused a significant upsurge in casualties [36], [10]. The World Health Organization indicates the number of fatalities from traffic crashes per year of about 1.35 million and 50 million injuries was recorded or an average of 3000 deaths/day and 30,000 injuries/day. Furthermore, post-crash medical care and maintenance of public facilities have also had a significant impact on the socio-economic landscape. This has elevated traffic safety to a prominent issue within the realm of public safety, severely affecting urban social stability and economic development. Precision analysis of traffic crash severity can identify dangerous sections of the road, effectively reducing the probability of traffic crashes, alleviating traffic congestion, and providing a theoretical basis for managers to implement targeted risk management measures. Moreover, emergency resource scheduling relies on credible traffic crash severity analysis, which would prospectively reduce secondary injuries caused by delayed rescue time [40], [6]. In addition, precision prediction of crash severity holds significance for the emergency warning functionality of autonomous driving systems.

Previous studies on crash severity analysis can be grossly divided into two categories: statistical methods and machine learning methods. The statistical methods, focus on analyzing historical traffic crash data to determine the correlation between risk factors and crash rates, and establishing a quantitative model capable of forecasting future crashes, such as the logit model [4], [11], ordered logit model [7], [29], [41], and ordered probit model [2]. Specifically, Lalika et al. [17] developed a Bayesian Logistic Regression model to identify significant factors influencing pedestrian fatalities and severe injuries. Hou et al. [12] used random parameters logit models to investigate crash-injury severities and clarified the out-of-sample prediction, the calculation of marginal effects, and temporal instability testing. Chen et al. [2] employed a random parameters bivariate ordered probit model to classify crash severity, achieving satisfying results. Statistical models are characterized by simple computing and high efficiency. However, statistical models rely on high-quality data during the training process, which usually show weakness in dealing with complex nonlinear data, resulting in the decline of prediction accuracy.

Machine learning is an advanced artificial intelligence algorithm that enables automatic analysis of interrelated data to extract valuable information, predict future trends, and facilitate independent learning and intelligent decision-making. Machine learning models are remarkably flexible in adjusting to novel data, allowing them to achieve superior performance when confronted with intricate, nonlinear, non-gaussian, and non-stationary relationships. As a result, they are broadly leveraged in analyzing the crash severity of traffic crashes. Typically, representative models include support vector machine (SVM) [28], gradient boosting decision tree (GBDT) [32], Random forest (RF) [34], multi-layer perceptron (MLP) [27], [24], convolutional neural network (CNN) [33], the Long Short-term Memory (LSTM) neural network [39], and ensemble models [26], [42]. Specifically, Wu et al. [32] presented an innovative GBDT model to explore the correlative impact of various risk factors on traffic crashes. Zhang and Abdel-Aty [38] proposes a bidirectional long short-term memory (LSTM) model with two convolutional layers to predict real-time crash potential on freeways, and proves that the proposed model can be successfully applied to another similar data set. Ma et al. [19] suggested a comprehensive analysis framework based on SSAE to predict the crash severity. In comparison with statistical models, machine learning models have a great advantage in processing complex and nonlinear relationships within traffic c data. The models often exhibit superior capabilities in feature learning and achieve better performance. Although machine learning models show advantages in shallow feature learning, they are still limited in revealing inner semantic information during model training. Furthermore, these models exhibit a lack of sensitivity towards noise data and have difficulty in handling imbalanced traffic crash datasets, which significantly diminish prediction accuracy.

Numerous research methods already exist in the field of crash severity analysis. However, there are still challenges in the accuracy of crash severity analysis models. On the one hand, the original features of traffic crash data are insufficient to describe the general patterns between crashes, and there are numerous inner features of the crash data yet to be explored. Inner feature learning [3], [18], [25] could enrich semantic information within the crash data, and enhance model generalization and adaptability, thereby boosting their classification efficacy. However, the traffic crash feature contains a wealth of semantic information, lacking effective and adequate development and utilization. On the other hand, there is an inherent imbalance in traffic crash data [43], [37]. Conventional models have a better performance in processing balanced data, however showing limitations in imbalanced data, consequently leading to precision decline. Simultaneously, previous models struggle to effectively extract and train the relationships in mixed linear and nonlinear traffic crash data. Therefore, addressing the imbalance of traffic crashes is crucial to improve prediction precision and the robustness of the model.

To address the above problems, this research proposes a data-enhanced double layer Stacking model using semantic understanding, named EnLKtreeGBDT, to predict traffic crash severities. The model aims to enhance traffic crash semantic learning of inner features and mitigate the impact of data imbalance to improve the prediction precision and robustness of the model. Firstly, the model primarily adopts the idea of enhanced learning to design a semantic enhancement module, extracting the inner semantics in various traffic crash features. Specifically, it contains a multi-dimensional feature derivation and a feature selection in which the multi-dimensional feature derivation is introduced to extract richer semantic information, and the feature selection is to filter out low-contributing features, accordingly enhancing the quality of the main features. Through analyzing the impact of various crash features, we can realize the analysis of the causes of traffic crashes, discover the potential laws of crashes, and provide a theoretical basis for accident prevention. Secondly, a data enhancement module, bases on data denoising and data migration, is proposed to effectively improve the imbalance in crash data. Through the data enhancement module, we can solve the inherent imbalance in the traffic crash data, alleviate the model deviation and improve the prediction performance of the model. Finally, to extract and train the relationships in mixed linear and nonlinear traffic crash data, a two-layer stacking model aggregating multiple linear and nonlinear classifiers is constructed to derive the final classification results.

The rest of this paper is organized as follows: Section 2 introduces the detailed composition of the dataset. Section 3 describes the proposed model EnLKtreeGBDT. Experimental results are provided in Section 4. Finally, the Section 5 includes the conclusions.

## 2 Data description

The crash severity data is collected from public data on road safety traffic crashes and vehicles in UK. The dataset includes 38 kinds of basic information from 2017 and 2018 and contains three categories of data: accident information, casualty information, and vehicle information. The details of the dataset are shown in Table 1.

**Table 1:** Basic data information.

Categories	Items	Description
Accidents	**Table:** Accident_Index Accident_Severity Police_Force Accident Date Accident Time Local_Authority_District Local_Authority_Highway Road_Type Speed_Limit Light_Conditions Weather_Conditions Road_Surface_Conditions Urban_or_Rural_Area	**Table:** Crash identification Crash severity The police force at the time of the crash Date of crash Time of Crash Local government area division in the UK The road network managed and maintained by the British local government The road type The speed limit Lighting conditions at the time of the crash Weather conditions at the time of the crash The surface of the road where the crash occurred Area where the crash occurred

Casualties	**Table:** Sex_of_Casualty Pedestrain_Location Pedestrain_Movement Car_Passenger	**Table:** The sex of casualty The relative position of the pedestrian and the vehicle at the time of the crash The movement, behavior or movement of pedestrians at the time of the crash The number of car passenger

Vehicles	**Table:** Vehicle_Reference Vehicle_Type Towing_and_Articulation Vehicle_Manoeuver Vehicle_Location Junction_Location Skidding_and_Overturning Hit_Object_in_Carriageway Hit_Object_off_Cariageway Purpose_of_Driver Sex_of_Driver Age_of_Driver Age_Band_of_Driver Engine_Capacity(CC) Propulsion_Code Left_Hand_Driver Age_of_Vehicle Driver_IMD_Decile Driver_Home_Area_Type Vehicle_Leaving_Carriageway Point_of_Impact	**Table:** Vehicle identification in the crash The vehicle type Towing and connection relationship between vehicles The manipulation behavior or action of the vehicle in the event of the crash The location or situation of the vehicle at the time of the crash The location of the intersection where the crash occurred Did the vehicle slip or roll over in crash The situation where the vehicle hits an object on the carriageway in crash When a vehicle hits an object outside the lane when it crashes The purpose of driver The sex of driver The age of driver The age band of driver The displacement of the vehicle engine The type of energy or power system used by the vehicle Does the driver drive with his left hand The age of vehicle Driver's socio-economic status Type of driver's residential area The situation where the vehicle leaves the carriageway in crash The location or part of the vehicle crash in traffic crash

The dataset is categorized into three different severities based on the severity of traffic crashes. The details of the crash severity are shown in Table 2.

**Table 2:** Crash severities.

Crash severity	Description	Frequency	Percent (%)
Class 1	Severe injury	1379	0.89
Class 2	Minor injury	26066	16.96
Class 3	No injury	126264	82.15
Overall	-	153709	100.00

Class 1 denotes Severe injury which account for 0.89% of total crashes. Class 2 represents Minor injury which respectively account for 16.96% of all crashes. Class 3 indicates No injury and the corresponding number of samples takes up more than half (82.15%) of all crash records. In the analysis of crash severity in the research, the experiment of mult-classification with three classes is applied to validate prediction precision.

## 3 Methodology

**Figure 1:**
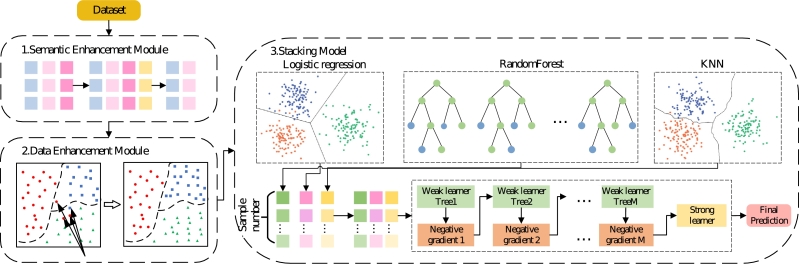
EnLKtreeGBDT for traffic crash severity prediction.

EnLKtreeGBDT for crash severity analysis as shown in Fig. 1, contains three fundamental components: semantic enhancement module, data enhancement module, and Stacking ensemble model. Firstly, to address the issue of insufficient semantic information learning, a semantic enhancement module is constructed to effectively extract implied semantic information inherent in multi-dimensional features, enhancing the characterization ability of the model. Secondly, we develop a data enhancement module to solve the problem of imbalance in traffic crash datasets by data denoising in large samples and increase the small samples size, which would minimize the adverse impact of imbalanced data on model precision and result in good generalization. Finally, a semantic and data-enhanced two-layer Stacking model consisting of linear and nonlinear learners, EnLKtreeGBDT, is designed to effectively predict the crash severity of traffic crashes.

### 3.1 Semantic enhancement

The traffic crash has complex and diverse risk factors. The original crash data exploration is insufficient to meet the inner semantic feature learning, such as the inherent periodicity of temporal features. The limitation would lead to a reduction in the accuracy of the prediction models. Comprehensive understanding and exploration of the initial data can effectively strengthen the semantic associations among features. That is, inner semantic feature learning would help to identify and utilize vital feature information, meanwhile, improve the capacity of the prediction model to deal with complicated and changeable traffic environments. Therefore, a semantic enhancement module is designed to explore valuable inner semantic information in traffic crash data.

The semantic enhancement module applied to obtain highly informative semantic features is composed of two parts: the feature derivation and the feature selection. To enrich the semantic information in traffic crash data, the feature derivation is leveraged to construct multi-level semantic information, which can be summarized into three types of features: temporal features, crash safety features, and collinear features. Then, the feature selection based on the Classification and Regression Tree (CART) is introduced to select the high-impact features by filtering out low-contribution irrelevant features.

#### 3.1.1 Feature derivation

**Figure 2:**
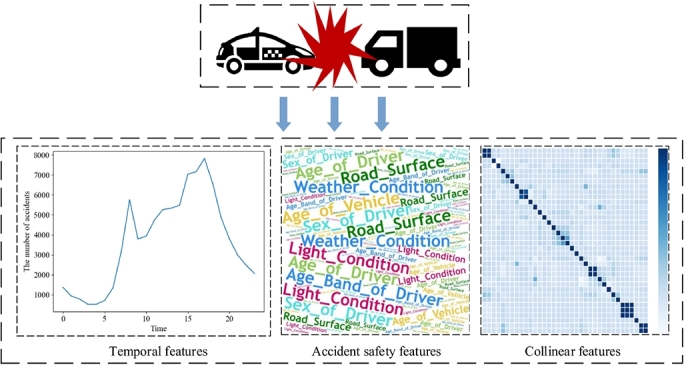
Three types of traffic crash features for feature derivation.

The quality of traffic crashes data significantly impacts on the prediction performance, which determines the upper limit of the information that the model can learn. In contrast, the optimal proposed models and algorithms can only approach this learning upper limit. Therefore, it is extremely necessary to optimize the data features to reflect the underlying laws of traffic crashes for prediction performance improvement. Feature derivation refers to the process of extracting relevant features from raw data through mathematical or statistical methods, which is commonly used to identify and extract significant features for better data understanding. In this section, we conclude three types of traffic crash features: temporal features, crash safety features, and collinear features for derivation to enhance the semantic understanding of crash data, thus to improve prediction precision. This is shown in Fig. 2.

(1) Derivation of temporal features

Temporal features of traffic crashes are crucial risk factors in the analysis of crash severities. Identifying the distribution pattern of traffic crashes over a period of time would be beneficial to develop efficient risk mitigation strategies. However, the collected data suffers from issues of non-discretization and single feature-based temporal features, which would ignore inherent features in crash data, such as periodicity. Therefore, we choose the temporal features for derivation to enhance semantic information and further explore the distribution of traffic crashes in the time dimension.

**Figure 3:**
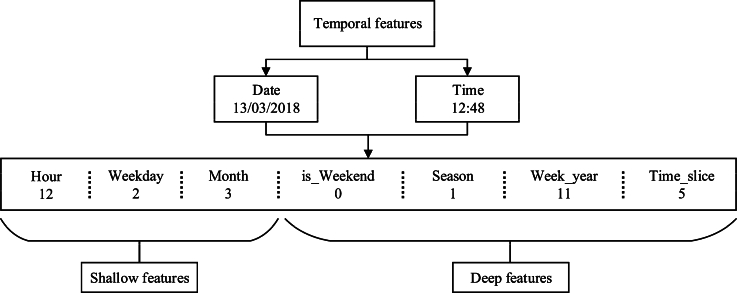
Temporal features derivation.

As shown in Fig. 3, we employ a deep and shallow combination strategy to derive the temporal features, enriching semantic information. The shallow features are derived from the intuitive time series in traffic crash data, it consists of Hour, Weekday, and Month which are the basic temporal features. We further derive the temporal features of is_Weekend, Season, Week_year and Time_slice as inherent features to reflect the crash periodicity, where the is_Weekend refers to the occurrence of the crash is weekend or not, Week_year refers to the week number of a year, Time_slice refers to the time slice in one day, for example, we separate one day to 8 slices, 12:48 belongs to the fifth slice. Through deep and shallow combination strategy, it helps to explore the features of accident-prone periods.

(2) Derivation of crash safety features

Crash safety features are highly correlated with the causes of crashes, which determine the crash severity classification to a great extent. Deriving crash safety features would help to provide more abundant, beneficial, and diversified semantic information to improve the feature representation capacity of the model. Considering the problem of large numerical gaps among various features, we select the standardized features with smaller gaps as much as possible to derive the crash safety features, then use the polynomial derivative method to obtain multidimensional semantic features. The feas_add and feas_mult are features that generated by polynomial derivation, as in Equation (1) and Equation (2).

(1) feas_add=feas[i]+feas[j]

(2) feas_mult=feas[i]×feas[j]

where feas_add is a new feature derived from the addition polynomial, feas_mult is a new feature derived from multiplication polynomials, feas[*i*] and feas[*j*] are any of two different dimensional features in the crash safety features, *n* is the total number of crash safety features.

(3) Derivation of collinear features

**Figure 4:**
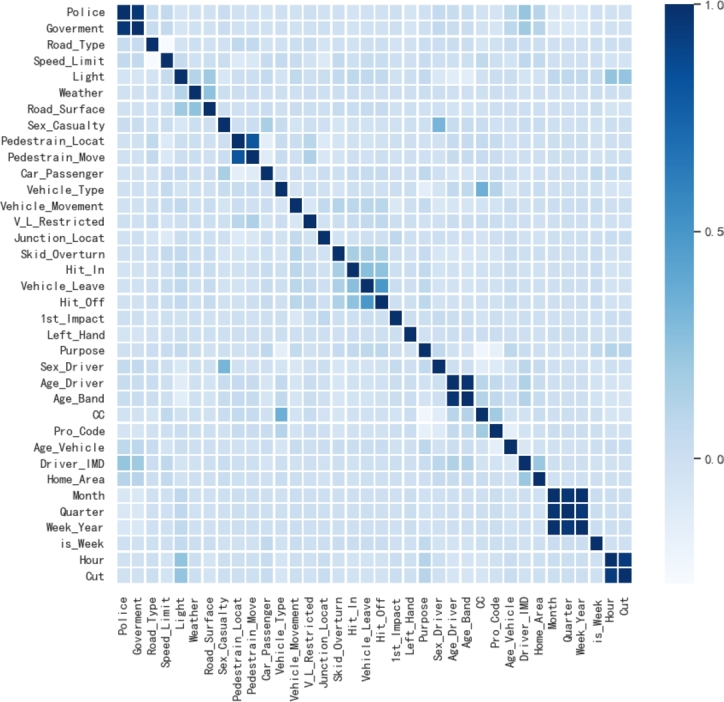
Feature correlation analysis.

Collinear features are of high linear correlation that would cause multicollinearity problems in data analysis and reduce the stability and accuracy of the prediction model. Therefore, it is necessary to detect and eliminate collinear features to improve the performance and robustness of the model. To solve this problem and ensure the validity of semantic information of crashes, we employ the Pearson correlation coefficient to analyze the correlation among features and obtain collinear features. As shown in Fig. 4, the Engine_Capacity (CC) and the Vehicle_Type are collinear features. The crash dataset in this research shows that motorcycles of 125cc and under are usually more prone to crashes than 125cc to 500cc motorcycles. Deriving features can better reflect the performance of the vehicle, so as to more accurately predict the severity of traffic crashes.

Then we utilize the feature cross-derivation to generate more meaningful features from correlated, different features to mitigate colinearity and improve the prediction precision. We select the maximum and minimum values in the features as the cross-derived parameters, as shown in Equation (3) and Equation (4). The maximum and minimum can better reflect extreme situations and indicate the range of data. For example, weather and light are experimental correlated features, adverse weather, and poor light may limit the vision of the driver, which would result in potential safety hazards. Therefore, to better represent the extreme situation, the maximum and minimum of weather and light variables are chosen to be derived. In addition, we introduce the average value between correlated features to narrow the range of multi-dimensional features, and lead to a better representation of the overall situation of traffic crashes, as shown in Equation (5).

(3) fea[get_max]=max⁡{feas_c[i],feas_c[j]}

(4) fea[get_min]=min⁡{feas_c[i],feas_c[j]}

(5) $$\mathrm{fea}[\mathrm{get}\_\mathrm{mean}]=\frac{\mathrm{feas}\_c[i]+\mathrm{feas}\_c[j]}{n}$$

where *feas_c* is the selection of a subset of collinear features. The *fea[get_max]*, *fea[get_min]*, and *fea[get_avg]* are the features generated by the extreme value and average value.

#### 3.1.2 Feature selection

Feature derivation is helpful to further enrich multiple crash features, but it also generates some irrelevant features which would increase model complexity and reduce training efficiency. For the purpose to improve the quality of primary features, we employ feature selection [15], [30], [8], [23] to filter irrelevant features.

Feature selection is a process that removes redundant or irrelevant variables in order to find a set of relevant features that better describe our data, and ideally, results in a more robust prediction performance [5]. We explore a Classification and Regression Trees (CART) algorithm to filter low-correlation features to enhance affect data quality and identify features with high information gain to improve model prediction performance.

The CART is based on the Gini index for feature selection. The Gini index is an indicator denoting the purity of the dataset, which can measure the contribution of features. Assuming that the proportion of class k in sample set D is $p_{k}(k=1,2,\ldots ,y)$, where y is the total classes, the Gini index of sample set D is calculated as Equation (6).

(6) $$\mathrm{Gini}(D)=\sum_{k=1}^{y}\sum_{k\neq k^{\prime }}p_{k}p_{k^{\prime }}=1-\sum_{k=1}^{y}p_{k}^{2}$$

Suppose discrete feature a has V possible values as $\left\{a^{1},a^{2},\ldots ,a^{V}\right\}$. If we use feature a to divide the sample set D, it will generate V branch nodes, where the v-th branch node contains all samples on feature a valued $a^{v}$ in sample set D, denoted by $D_{a}^{v}$. Equation (7) shows the specific calculation process of the Gini index of feature a.

(7) $$\mathrm{Gini}\_\mathrm{index}(D,a)=\sum_{v=1}^{V}\frac{\left|D_{a}^{v}\right|}{\left|D\right|}\mathrm{Gini}(D_{a}^{v})$$

**Figure 5:**
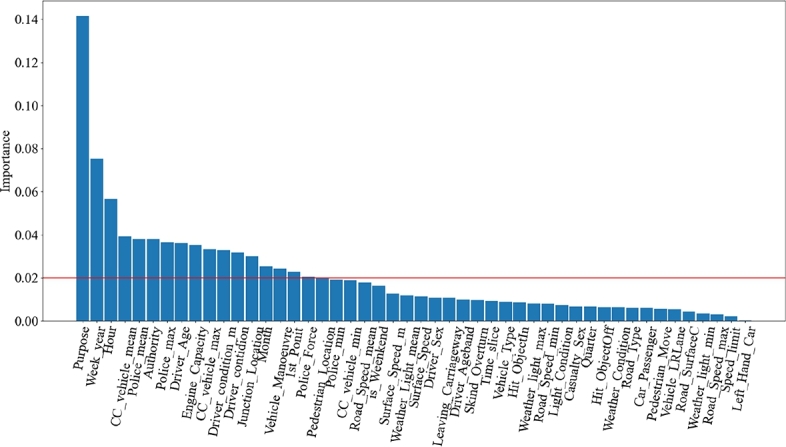
Feature contribution diagram.

Fig. 5 shows the contribution of each feature calculated by CART. We rank the contributions by size and select high information gain features with a contribution greater than 0.02. According to the contribution of each feature, we select the first 17 features for model training.

### 3.2 Data enhancement

High-quality data are critical for validly monitoring progress toward initiatives related to road traffic crash prevention [14]. Traffic crash data has an inherent imbalance which can affect the data quality [31]. During the model training, it commonly tends to learn large sample data and ignores the learning of small sample data, which will lead to the decline of model prediction accuracy. Therefore, alleviating data imbalance would be a great step to optimize the model training process and improve the model prediction accuracy.

For the purpose to enhance the proportion of small sample data in the overall data, we design a data enhancement module, based on the data denoising and data migration, by eliminating noise in large samples and increasing the small samples training data to change the distribution of the data set. It is expected to alleviate the reliance of the classification model on large sample data, strengthen the model's learning ability of small sample data, and ultimately alleviate the data imbalance problem.

#### 3.2.1 Data denoising

**Figure 6:**
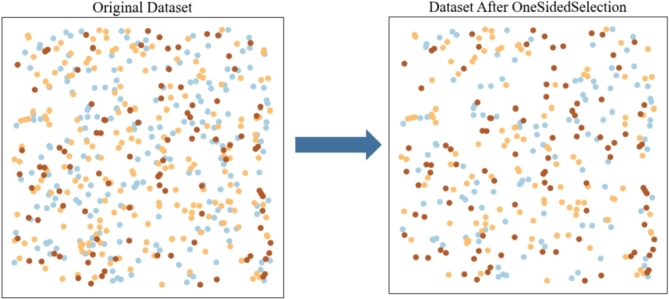
Data processing based on OSS.

Kubat et al. [16] propose the One-Sided Selection (OSS) that uses K-Nearest Neighbors (KNN) and Tomek links to improve the prediction accuracy of the model by removing low-quality subsets of samples. It is based on a rule to select some large samples called noise and delete them to alleviate the problem of data imbalance. We employ a two-step approach to address the issue of noise data and imbalanced data which affect model performance. Firstly, we use KNN to obtain the most representative data from small sample data. Secondly, Tomek links is applied to remove redundant data and noise data that are easy to cause misclassification. The approach has a better performance in reducing data noise and balancing traffic crash data. As depicted in Fig. 6, the application of OSS has resulted in reduced overall sample size, alleviating the impact of large sample classes on smaller ones.

To obtain the most representative data from small data, we employ the KNN, which is a basic machine learning algorithm using K closest labeled examples to classify or perform regression on unlabeled data points, to identify the data pairs with tomek links relationship. Specifically, to select representative sample data, we use Equation (8) to calculate the K nearest neighbors of each sample and divide the sample into 2 parts: Same class neighbors and Different class neighbors. And, we classify different class neighbors into internal samples and boundary samples according to the proportion of the small sample in the nearest neighbors of each sample to provide initial data for Tomek links.

Experimented by trial and error, we select K=6 in this research.

(8) $$d=\sqrt{\sum_{i=1}^{n}{\left(x_{i}-y_{i}\right)}^{2}}$$

Tomek links is a method used to remove samples located near the decision boundary that can cause confusion. Firstly, we calculate K nearest neighbors of each boundary sample to determine whether there is a Tomek links relationship between it and the small sample. We call the sample pairs with Tomek links relationship as Tomek links pairs. Secondly, to decline the large sample size, we use the Tomek links to eliminate the large sample data from the Tomek links pairs. By this method, we can reduce the large sample size and increase the proportion of small samples in the overall data to alleviate the impact of data imbalance on model performance.

**Figure 7:**
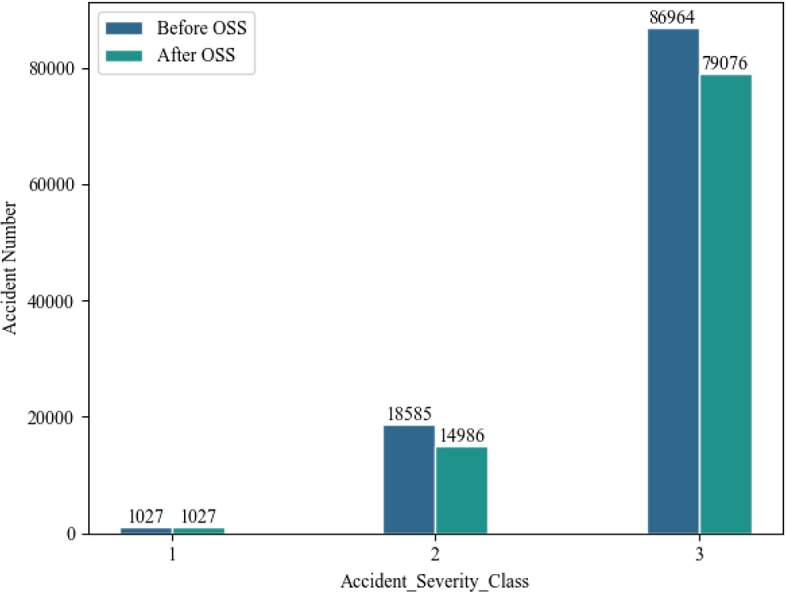
OSS processing results.

Fig. 7 illustrates the changes in data before and after utilizing OSS processing. It shows that the dataset of this research undergoes a 10.78% reduction in overall data size after being processed by OSS. Specifically, the Class 3 sample size is reduced by 9.07% and the Class 2 sample size is declined by 19.36%, while the Class 1 sample size is not changed. It is worth noting that the data distribution after OSS processing is 0.96: 14.6: 84.44, which is similar to the original distribution. It indicates that OSS is capable of reducing the large sample size by eliminating noise samples. At the same time, it is a great step toward facilitating the precision and robustness of the traffic crash prediction model.

#### 3.2.2 Data migration

Although the size of large sample data has been declined after introducing the OSS, there is still an extremely serious imbalance in the traffic crash dataset. Therefore, we introduce the method of Enhanced Data Migration, which incorporates Random Over-Sampling, to further mitigate the problem of imbalanced traffic crash data. This method enhances the ability of model to learn from small sample data, leading to improved prediction precision and robustness. Firstly, we introduce the Class 1 samples in the 2017 UK road safety crash dataset enhanced by Semantic enhancement. Secondly, Random Over-Sampling is applied to eliminate the problem of imbalanced data and remove the reliance of the prediction model on large sample data.

To completely address the problem of imbalanced datasets, Random Over-Sampling (ROS) technique [20] is proposed to balance the class distribution by generation synthetic samples of the minority class. The method randomly selects instances from the small sample and replicates them, effectively increasing their representation in the dataset. ROS consists of two main steps. Firstly, the minority class samples are randomly chosen with replacement, allowing for duplicate instances. Secondly, these selected samples are duplicated and added to the original dataset. By repeating this process, the minority class is oversampled, resulting in a more balanced distribution of classes.

During model training, the dataset is classified into two separate sets: the training set and testing set, and the ratio between the two is 6:4. As shown in Table 3, after classifying the dataset, the Class 1 sample size accounts for only 1.06% of the total sample, which is similar to the proportion of the original data. By introducing the Class 1 samples in the 2017 UK road safety crash dataset enhanced by Semantic enhancement, the proportion of small sample data is further increased. It can be seen from Table 3 that after Data Migrating, the proportion of Class 1 samples in the entire training set reached 2.44%. Simultaneously, the Random Oversampling technique is employed to augment the sample size of Class 1 and Class 2, aligning them with the sample size of Class 3. The data enhancement module has a great significance to solving the problem of imbalance data.

**Table 3:** Changes in different classes of sample in the training set.

Crash severity	Original		After Data Migration		After Random Over-Sampling	
	Frequency	Percent (%)	Frequency	Percent (%)	Frequency	Percent (%)
Class 1	607	1.06	1419	2.44	47664	33.3
Class 2	8975	15.68	8975	15.46	47664	33.3
Class 3	47664	83.26	47664	82.10	47664	33.3
Overall	57249	100	58058	100	142992	100

### 3.3 Double layer stacking model

In the complex urban traffic environment, crash data show a complex linear and nonlinear multilevel relationship. Traditional crash prediction models, such as the generalized linear regression model, are incapable of taking into account multilevel data structure [13]. Stacking [35], [1] is an ensemble learning method to integrate multiple classifiers usually with a two-layer structure. In the first layer, several base classifiers are integrated to extract valid features. In the second layer, a meta-classifier is trained based on the outputs of base classifiers and calculates the final prediction results of the crash severity. To improve the ability of the model to learn linear and nonlinear data, we establish a two-layer ensemble model based on the Stacking method.

The selection of base classifiers is of great significance to the performance of the Stacking model. It is necessary not only to ensure the accuracy of the classification effect, but also to ensure the diversity of algorithms. We have chosen three different base classifiers to be used in the first layer of the Stacking model: the linear classifier (Logistic Regression), the ensemble classifier (Random Forest), and the nonlinear classifier (K-Nearest Neighbors). The Logistic Regression (LR) model focuses on analyzing historical traffic crash data to determine the linear correlation between risk factors and crash severity, and achieve a higher prediction performance of the model. The Random Forest (RF) is an efficient ensemble model that integrates multiple decision trees to complete classification and prediction tasks. The KNN is a nonlinear classifier that effectively distinguishes different samples. Firstly, KNN will compare the training data with the test data to find the K most similar samples. Secondly, the class of the testing data is determined according to the voting method. The choice of the three algorithms can consider simultaneously local and global features, which helps to improve the accuracy and robustness of the urban crash prediction model.

The selection of meta-classifier should have appropriate complexity, robustness, inductive bias, and efficiency, to prevent model overfitting to a great extent. GBDT is an ensemble learning model that fits the negative gradients by iteratively integrating weak learners. It has the advantages of decision tree complexity and robustness, and can decline the degree of model overfitting by limiting parameters such as the depth of the decision tree. In addition, GBDT adopts a gradient boosting algorithm, which can further facilitate the prediction performance of the model and be better used for complex classification tasks.

**Figure 8:**
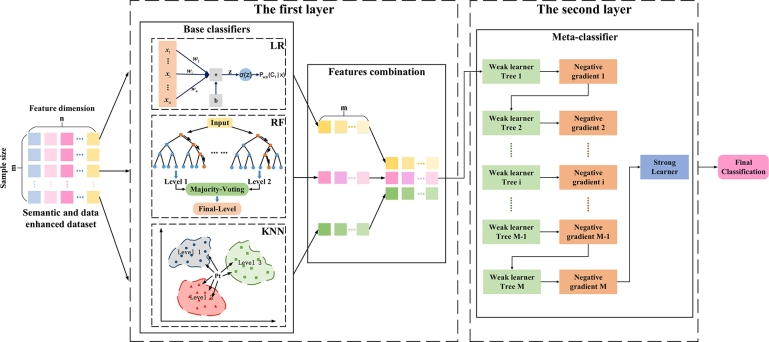
LKtreeGBDT.

As shown in Fig. 8, we select LR, RF, and KNN as the base classifiers to effectively extract features in the first layer, and GBDT as the meta-classifier in the second layer to establish a Stacking model known as LKtreeGBDT, which has a double-layer structure.

During model training, the dataset is classified into two separate sets: the training set and testing set, and the ratio between the two is 6:4. We adopt the five-fold cross-validation method to overcome the over-fitting problem. Algorithm 1 shows pseudo codes of the Stacking method proposed in the research.

**Algorithm 1:**
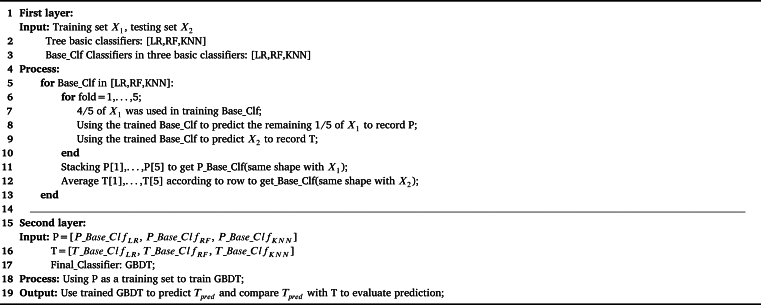
Stacking algorithm.

## 4 Experimental results and discussion

### 4.1 Experimental setup

#### 4.1.1 Dataset

The crash severity data collected from the UK road safety crashes. In this paper, we select 153709 samples of data with 38-dimensional features to analyze the traffic crash severity. The detailed dataset is shown in Table 1 and Table 2.

#### 4.1.2 Parameters for the stacking model

Table 4 presents specific parameter settings for the stacking model, which based on the recommendations of Tang et al. [26] and Probst et al. [22], and all parameters of our model are set by trial and error to yield an optimal structure.

**Table 4:** Parameters setting in Stacking model.

Stacking structure	Model	Parameters setting
First layer	LR	C=0.1, max_iter=3000, n_jobs=8, random_status=1412
	RF	n_estimators=100, max_features=sqrt, max_samples=0.9, n_jobs=8
	KNN	n_neighbors=10, n_jobs=8

Second layer	GBDT	n_estimators=100, max_features=16, random_status=4869

#### 4.1.3 Evaluation indicator

In this research, we select Precision, Accuracy, F1-score, and weighted_avg as the performance evaluation indicators of the model. The weighted_avg is a weighted average, which can evaluate the overall performance of the model. Equation (9) to Equation (14) show the specific calculation of the evaluation index.

(9) $${\mathrm{precision}}_{k}=\frac{P_{kk}}{\sum_{i=1}^{3}P_{ik}}$$

(10) $$\mathrm{Precision}=\frac{\sum_{i=1}^{3}{\mathrm{precision}}_{i}}{3}$$

(11) $$\mathrm{Accuracy}=\frac{\sum_{i=1}^{3}P_{ii}}{\sum_{i=1}^{3}\sum_{j=1}^{3}P_{ij}}$$

(12) $$f1-{\mathrm{score}}_{k}=2\times \frac{\frac{P_{kk}}{\sum_{i=1}^{3}P_{i1}}\cdot \frac{P_{kk}}{\sum_{i=1}^{3}P_{1i}}}{\frac{P_{kk}}{\sum_{i=1}^{3}P_{i1}}+\frac{P_{kk}}{\sum_{i=1}^{3}P_{1i}}}$$

(13) $$F1-\mathrm{score}=\frac{\sum_{i=1}^{3}f1-score_{i}}{3}$$

(14) $$\mathrm{weighted}\_\mathrm{avg}=\sum_{i=1}^{3}\frac{f1-score_{i}\times {\mathrm{support}}_{i}}{\mathrm{support}}$$

where $P_{ij}$ is the number of samples with a true value of *i* and a predicted value of *j*, and *support* represents the entire sample size (Table 5).

**Table 5:** Interpretation of symbols.

		True Value		
		Class 1	Class 2	Class 3
Predicted Value	Class 1	P_11_	P_21_	P_31_
	Class 2	P_12_	P_22_	P_32_
	Class 3	P_13_	P_23_	P_33_

### 4.2 Comparison of prediction performance

**Figure 9:**
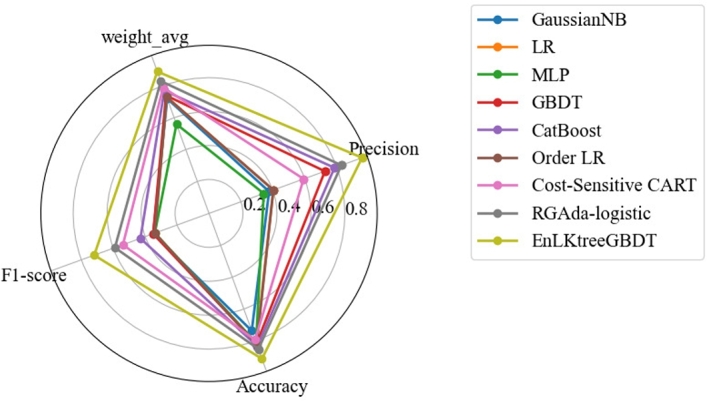
Comparison of model results.

To evidence the performance of the proposed Stacking model, EnLKtreeGBDT, is compared with eight traditional classification methods: the GaussianNB model, the LR model [9], the MLP model [27], [24], the GBDT model [32], the CatBoost model [21], the Order LR model [7], the Cost-Sensitive CART model [44], and the RGAda-logistic model [26]. To ensure a fair comparison, all the models are trained based on the same training set and tested on the same testing set. The prediction results of different models for the traffic crash severity are shown in Fig. 9 and Table 6.

**Table 6:** Comparative experiment.

Model	Precision	Accuracy	F1-score	weighted_avg
GaussianNB	37.67%	73.66%	0.35	0.72
LR	40.33%	81.51%	0.34	0.73
MLP	34.33%	81.15%	0.34	0.56
GBDT	72.67%	81.79%	0.35	0.74
CatBoost	78.67%	83.39%	0.43	0.77
Order LR	40.33%	82.26%	0.34	0.73
Cost-Sensitive CART	59.33%	79.35%	0.54	0.79
RGAda-logistic	83.00%	85.14%	0.59	0.83
**EnLKtreeGBDT**	**96.00%**	**91.23%**	**0.72**	**0.89**

Fig. 9 depicts the predictive performance of the EnLKtreeGBDT model and the benchmark models on the four indicators intuitively. It can be observed that the EnLKtreeGDBT model outperforms the eight benchmark models in the four indicators of predictive performance. The specific data are presented in Table 6. The experimental results show that the maximum increasements of the nine models observed in Precision, Accuracy, F1-score, and weighted_avg are respectively 61.67%, 17.57%, 0.38 and 0.33, while the minimum are 13.00%, 6.09%, 0.13 and 0.06. It validates the advantage of the Stacking model, EnLKtreeGBDT, to learn from complex nonlinear data. Meanwhile, it can be seen that the EnLKtreeGBDT has a higher prediction precision in the complex urban traffic environment than the benchmark models.

### 4.3 Performance verification of semantic enhancement module

To evaluate the efficacy of the semantic enhancement module, it is integrated into all the models discussed in this research, including the eight benchmark models, and experiments are conducted on UK road safety crash data. The results are shown in Table 7.

**Table 7:** Performance verification of the data enhancement module.

Model	Add Semantic Enhancement Module				Original Model			
	Precision	Accuracy	F1-score	weighted_avg	Precision	Accuracy	F1-score	weighted_avg
GaussianNB	35.66%	79.08%	0.32	0.72	37.67%	73.66%	0.35	0.72
LR	46.00%	81.54%	0.30	0.73	40.33%	81.51%	0.34	0.73
MLP	41.33%	81.53%	0.30	0.73	34.33%	81.15%	0.34	0.56
GBDT	67.33%	81.94%	0.33	0.74	72.67%	81.79%	0.35	0.74
CatBoost	81.67%	82.33%	0.43	0.76	78.67%	83.39%	0.43	0.77
Order LR	46.00%	81.55%	0.30	0.73	40.33%	82.26%	0.34	0.73
Cost-Sensitive CART	58.67%	81.60%	0.59	0.81	59.33%	79.35%	0.54	0.79
RGAda-logistic	89.33%	87.13%	0.68	0.85	83.00%	85.14%	0.59	0.83
**LKtreeGBDT**	**88.67%**	**88.24%**	**0.68**	**0.85**	**85.00%**	**85.96%**	**0.59**	**0.83**

Table 7 shows that the performances of the partial benchmark models and the Stacking model, LKtreeGBDT, have been significantly improved after integrating the semantic enhancement module. The MLP has exhibited a noteworthy increase in precision indicator, amounting to 20.39%, with a significant impact. In addition, the Stacking model, LKtreeGBDT without enhancement modules, has increased by 4.32%, 2.65%, 15.25% and 2.41% in the four indicators after adding the semantic enhancement module. However, most models have different degrees of reduction on the F1-score where the LR, MLP, Order LR models have declined 11.76%. For weighted_avg indicator, all models have demonstrated no significant increasements. These results underscore the effectiveness of the semantic enhancement module, revealing its pivotal role in enhancing the performance and robustness of the majority of models, albeit with potential variability across different model architectures. Importantly, the experiment illuminates the module's proficiency in extracting inner semantic information, thus substantiating its efficacy in model optimization.

**Figure 10:**
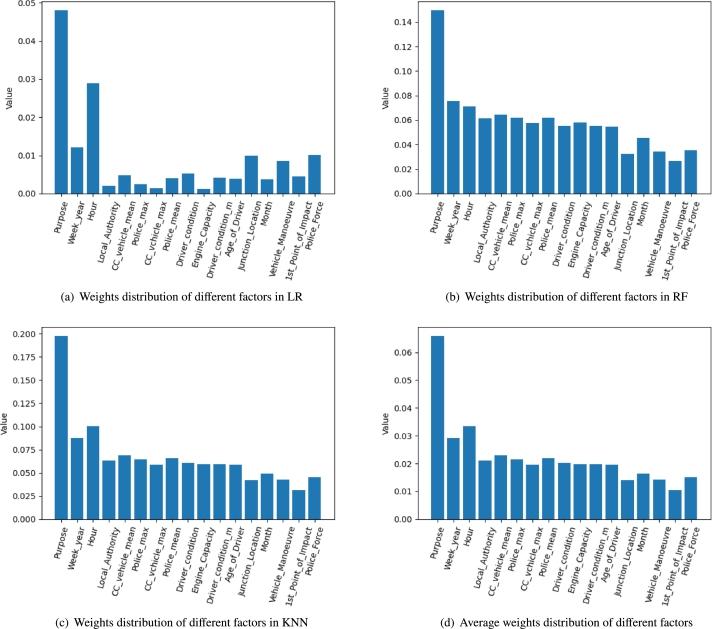
Contributions of each factor.

In order to evaluate the contribution of different features to the traffic crash severity, we extract the weights and average weights of each feature in the different base classifier as shown in Fig. 10. The 17-dimensional features in the Fig. 10 are the high-contribution features that we have selected based on the CART algorithm. Week_year, Hour, CC_vehicle_mean, Police_mean, Driver_condition, Driver_condition_m, and Month are derived features from the semantic enhancement module which make unique contributions in the three base classifiers. Fig. 10(a) shows the weights of different features in LR. It can be observed that Hour has a large weight, second only to Purpose. Fig. 10(b) and Fig. 10(c) show that each derived feature has relatively considerable weight to the traffic crash severity in RF and KNN. As can be seen from Fig. 10(d), the derived features have a weight that is not much different from the rest of the features except for Purpose. This verification underscores the pivotal role of the semantic enhancement module in optimizing the model training process and enhancing the efficacy of base classifiers in feature extraction. These findings highlight the module's potential to optimize the process of model training, thereby contributing to the overall robustness and efficiency of the predictive model.

### 4.4 Performance verification of data enhancement module

To highlight the impact of imbalanced data on the performance of model and verify the impact of the data enhancement module on model performance, we integrate data enhancement module into all models that contain a semantic enhancement module. The results are shown in Table 8.

**Table 8:** Performance verification of the data enhancement module where EnLKtreeGBDT is LKtreeGBDT based on semantic and data enhanced modules.

Model	Add Semantic and Data Enhancement Module				Add Semantic Enhancement Module			
	Precision	Accuracy	F1-score	weighted_avg	Precision	Accuracy	F1-score	weighted_avg
GaussianNB	36.33%	61.16%	0.53	0.66	35.66%	79.08%	0.32	0.79
LR	36.00%	42.42%	0.28	0.53	46.00%	81.54%	0.30	0.73
MLP	37.00%	58.85%	0.35	0.66	41.33%	81.53%	0.30	0.73
GBDT	39.00%	57.13%	0.36	0.65	67.33%	81.94%	0.33	0.74
CatBoost	45.33%	72.27%	0.48	0.75	81.67%	82.33%	0.43	0.76
Order LR	36.00%	42.45%	0.28	0.53	46.00%	81.55%	0.30	0.73
Cost-Sensitive CART	58.67%	84.89%	0.60	0.84	58.67%	81.60%	0.59	0.81
RGAda-logistic	91.66%	90.04%	0.71	0.89	89.33%	87.13%	0.68	0.85
**LKtreeGBDT**	**96.00%**	**91.23%**	**0.72**	**0.89**	**88.67%**	**88.24%**	**0.68**	**0.85**

Table 8 demonstrates the comparisons between the models that integrate semantic enhancement module and the models that add semantic and data enhancement modules. It can be seen that after integrating the data enhancement module, the four indicators of the Cost-Sensitive CART, RGAda-logistic, LKtreeGBDT models have improved. Specially, the LKtreeGBDT has improved 8.27%, 3.39%, 5.88% and 4.71% on these four indicators. These findings suggest the data enhancement module can significantly address the inherent imbalance in traffic crash data, decline the effect of imbalanced data on model performance, and optimize the prediction performance of complex models. In addition, compared to benchmark models with two enhancement modules, our model achieves the best performance, verifying the learning ability of the Stacking model on nonlinear data. Which underscores the robustness and adaptability of the EnLKtreeGBDT model in complex and dynamic urban road environments, and highlights its distinct advantages in the prediction of traffic crash severity.

However, it can be seen that the precision, accuracy and weighted_avg indicators parts of benchmark models exhibit a range of decline, with the highest decline reaching 44.50%, 47.98% and 27.40%, while the lowest showing 10.47%, 12.21% and 1.32%. This is because the data enhancement module generates a large number of duplicate samples which will interfere with the training of simple benchmark models. However the EnLKtreeGBDT model proposed in this paper can extract information that is effective for model training from complex data. This shows that the complex structure of stacking can be effectively integrated with the data enhancement module to solve the problem of data imbalance and improve the prediction performance of the model.

### 4.5 Ablation model

**Figure 11:**
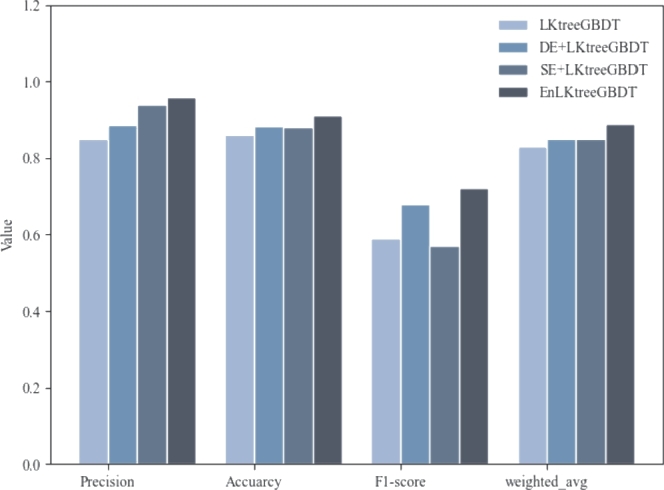
Comparisons of ablation experimental results where *DE* is the data enhancement module and *SE* represents the semantic enhancement module.

In this section, we design the ablation experiment to analyze the impact of the semantic enhancement module and data enhancement module on the prediction performance of the EnLKtreeGBDT model. Fig. 11 and Table 9 show the performance comparisons of the models after integrating the two enhancement modules separately.

**Table 9:** Ablation experiment.

Model	Precision	Accuracy	F1-score	weighted_avg
LKtreeGBDT	85.00%	85.96%	0.59	0.83
DE+LKtreeGBDT	88.67%	88.24%	0.68	0.85
SE+LKtreeGBDT	94.00%	87.91%	0.57	0.85
**EnLKtreeGBDT**	**96.00%**	**91.23%**	**0.72**	**0.89**

Fig. 11 shows that EnLKtreeGBDT is superior to the other ablation models in four indicators. Table 9 is evident that EnLKtreeGBDT has exhibited significant improvement in four indicators as compared to LKtreeGBDT, DE+LKtreeGBDT, and SE+LKtreeGBDT. The four indicators increased by the highest 15.66%, 6.13%, 22.03% and 7.23% respectively, whereas the lowest increases are noted at 8.27%, 3.39%, 5.88% and 3.49%. This confirms that the semantic enhancement module effectively explores inner correlation from the traffic crash data, enhances the semantic relationships among the features, and improves the prediction precision of the Stacking model.

It is worth noting that DE+LKtreeGDBT has 3.40% decline in F1-score indicator to LKtreeGBDT. However, compared to SE+LKtreeGBDT, the F1-score of EnLKtreeGBDT improves 4.88% by after integrating the data enhancement model. This shows that semantic enhancement model has a supportive effect on data enhancement model. Simultaneously, the data enhancement module demonstrates an effective approach to eliminating noise data from imbalanced traffic crash data. It is conducive to eliminating data imbalance, reducing model deviations, and ultimately improving the performance and robustness of the urban crash severity prediction model.

## 5 Conclusions

In this paper, a two-layer Stacking ensemble model called EnLKtreeGDBT, based on two enhancement modules, is developed and employed to predict the severity of crashes using UK road safety crash data. The dataset is classified into a training set and a testing set. Considering comprehensively the inner nature of traffic crashes and the specificity of the classification task, we use Precision Accuracy, F1-score, and weight_avg as evaluation indicators. We compare EnLKtreeGBDT with eight benchmark models to predict its effectiveness in complex urban road environments. Additionally, we design multiple experiments to evaluate the efficiencies of the semantic enhancement module, data enhancement module, and Stacking model.

The contribution of this research is mainly summarized from the following three aspects: (1) Suggest a semantic enhancement module to reveal inner semantic information among various features, improving the quality of feature information. Through feature selection, low-contribution features are filtered out to enhance the contribution of the main features and improve the prediction precision of the model. By analyzing various crash factors, it helps reveal the underlying mechanisms of crashes and identify similarities among traffic crashes, thereby providing a scientific basis for the formulation of urban road policies. (2) Design a data enhancement module that addresses the imbalance in crash dataset, which achieves by decreasing large sample sizes and increasing the ratio of small samples. By this module, we decline the impact of imbalanced data on the prediction performance of the model to a great extent, alleviate the bias of the model. (3) Establish a two-layer Stacking ensemble model that employs three base classifiers, LR, RF, and KNN, for feature extraction. GBDT is selected as the second-layer meta classifier. This ensemble learning method offers the flexibility of using different base classifiers and meta classifier to improve the overall accuracy of the predictive model.

However, given the complexity and variability of traffic crashes, the study in this paper has inherent limitations: (1) The study utilizes public data on road safety traffic crashes and vehicles in UK. Future study should consider the integration of multi-data fusion techniques, exploring geospatial data such as remote video, street view images, social media, etc., combined with traditional traffic accident data, to further delineate the road risk environment and enrich the data sources for traffic crash research. (2) The process of identifying risk factors in road traffic accidents is a complex undertaking. While some of the deep semantics of features have been analyzed, there remain a large number of risk factors that have not yet been identified. Further study is imperative to fully comprehend the factors that contribute to road traffic accidents. (3) Stacking is an ensemble learning approach that combines multiple classifiers. This ensemble learning method offers the flexibility of using different base classifiers and meta-classifier to improve the overall accuracy of the predictive model. Further research could focus on the selection of different learners to improve model performance. Furthermore, it is recommended that future research should also explore the spatiotemporal dimensions of traffic crash factors. This area of research will contribute towards a more comprehensive understanding of the complex dynamic features involved in traffic crashes.

The analysis of crash causes and classification of traffic crash severity contribute to identifying hazardous road sections, thereby reducing the probability of traffic collisions and environmental traffic congestion. Additionally, precise prediction of traffic severity facilitates timely allocation of rescue resources and police deployment, while also holding crucial significance for optimizing emergency warning functionality in autonomous driving systems.

## CRediT authorship contribution statement

**Di Yang:** Writing – review & editing, Validation, Supervision, Resources, Project administration, Methodology, Formal analysis, Conceptualization, Software. **Tao Dong:** Validation, Methodology, Investigation, Data curation, Visualization, Writing – original draft, Writing – review & editing. **Peng Wang:** Funding acquisition, Resources, Supervision.

## Declaration of Competing Interest

The authors declare no potential conflicts of interest with respect to the research.

## Data Availability

All dataset and associated code files are available from https://github.com/123dongtao/UK-RSCrash.
